# Brain-Derived Neurotrophic Factor Expression in Patients with Acute Pulmonary Embolism Compared to the General Population: Diagnostic and Prognostic Implications

**DOI:** 10.3390/jcm11174948

**Published:** 2022-08-23

**Authors:** Mihai Ștefan Cristian Haba, Ionuț Tudorancea, Cosmin Teodor Mihai, Viviana Onofrei, Irina Iuliana Costache, Antoniu Octavian Petriș, Laurențiu Șorodoc

**Affiliations:** 1 Department of Internal Medicine I, Faculty of Medicine, University of Medicine and Pharmacy “Grigore T. Popa”, 700115 Iasi, Romania; 2 Department of Morpho-Functional Sciences II-Physiology, Faculty of Medicine, University of Medicine and Pharmacy “Grigore T. Popa”, 700115 Iasi, Romania; 3 Advanced Research and Development Center for Experimental Medicine (CEMEX), University of Medicine and Pharmacy “Grigore T. Popa”, 700115 Iasi, Romania; 4 Department of Internal Medicine III, Faculty of Medicine, University of Medicine and Pharmacy “Grigore T. Popa”, 700115 Iasi, Romania

**Keywords:** pulmonary embolism diagnosis, BDNF, D-dimers

## Abstract

(1) Background: Pulmonary embolism (PE) is a severe condition, representing the third most important cardiovascular cause of death after myocardial infarction and stroke. Despite the use of clinical pre-test probability scores, D-dimer measuring, and computer tomography pulmonary angiography (CTPA), PE diagnosis remains a challenge. Brain-derived neurotrophic factor (BDNF) is the most important member of the neurotrophin family, which has also been shown to be involved in the physiopathology of cardiovascular conditions such as heart failure and myocardial infarction. In this study, we aimed to assess the BDNF expression in patients with acute PE compared to the general population, and to also investigate its diagnostic and prognostic role. (2) Methods: We conducted a single center prospective study, which included 90 patients with PE and 55 healthy volunteers. Clinical and paraclinical parameters, together with plasma levels of BDNF, were evaluated in all patients after admission. (3) Results: The plasma levels of BDNF were significantly lower in the PE patients compared with the control group (403 vs. 644 pg/mL, *p* < 0.001). ROC analysis revealed an AUC of 0.806 (95% CI 0.738–0.876, *p* < 0.001) and a cut-off value of 564 pg/mL, which associated a sensitivity of 74.4% and a specificity of 78.2% for PE. Low BDNF levels also correlated with prognostic markers of PE, such as PESI score (*p* = 0.023), NT-proBNP (*p* < 0.01), right ventricular diameter (*p* = 0.029), and tricuspid annular plane systolic elevation (*p* = 0.016). Moreover, we identified a decreased BDNF expression in patients with high-risk PE (*p* < 0.01), thrombolytic treatment (*p* = 0.01), and patients who died within 30 days (*p* = 0.05). (4) Conclusions: Our study revealed that plasma BNDF is significantly lower in patients with PE when compared with the general population, and may be considered as a promising biomarker in complementing the current diagnostic tools for PE. Furthermore, low levels of BDNF might also be used to predict a poor outcome of this condition.

## 1. Introduction

Pulmonary embolism (PE) is a common and severe cardiovascular condition with an incidence ranging from 39 to 115 cases per 100,000 individuals [1]. After myocardial infarction and stroke, PE represents the third most important cause of vascular death, with mortality rates of 8% in patients with proper therapeutic management, and up to 30% in patients with delayed diagnostic and treatment [2]. According to current registries and observational studies, only 7% of patients who died early after hospital admission were appropriately diagnosed with PE before death [3]. Therefore, to reduce the high rate of life-threatening complications, the diagnosis of PE is of paramount importance, particularly in the first hours after hospital admission.

The main diagnostic tool for PE is computed tomography (CT) pulmonary angiography (CTPA), which has a sensitivity of 83% and a specificity of 96%. CTPA examination should be performed in the context of clinical judgement, as it has only a 60% negative predictive value if PE pre-test probability is high, and a 58% positive predictive value in patients with low PE pre-test probability [4]. However, CTPA also has limitations regarding radiation exposure and iodine contrast usage, and is contraindicated in certain groups at risk for developing PE, such as pregnant and postpartum women, and severe renal failure patients.

Although D-dimers are the most used biomarker to increase the pre-test probability of PE, they have been shown to have a negative predictive value > 95%, but with poor positive predictive values (67.6%) [5,6]. Moreover, in conditions which are proved to be linked with PE (cancer, pregnancy, hospitalized patients), plasma D-dimer levels are pathological, thus increasing the number of false positive results [7,8]. Other cardiac or vascular biomarkers, such as troponin, N-terminal pro b-type natriuretic peptide (NT-proBNP), or copeptin, have been shown to have poor diagnostic utility, but can be used as predictors of poor outcome or death [3].

Brain-derived neurotrophic factor (BDNF) is the most abundant endogenous factor of the neurotrophin family, and in the adult heart, it modulates contractility, neoangiogenesis, cell apoptosis, and survival [9,10]. Therefore, several attempts have been made to use circulating BDNF as a biomarker for the diagnosis and severity of cardiovascular diseases, mainly focused on coronary artery disease [10,11,12]. BDNF concentrations have also been found to be altered in pulmonary hypoxia, leading to the promotion of pulmonary artery smooth muscle cell proliferation and nitrous oxide (NO) production [11].

In this study, we aimed to investigate the expression of BDNF in PE patients compared with the general population. Furthermore, we assessed its diagnostic utility compared with D-dimers in our study cohort. By assessing the prognostic risk scores, clinical and paraclinical markers, and 30-day mortality, we also evaluated if BDNF may have a predictive value for the poor outcome in PE patients.

## 2. Materials and Methods

### 2.1. Study Design and Population

We conducted a prospective case-control study that evaluated 90 consecutively-enrolled patients with acute pulmonary embolism, diagnosed by CTPA, and admitted in the Cardiology Clinic of the St. Spiridon Emergency County Hospital (Iași, Romania) between June 2021 and April 2022. The control group included 55 sex- and age-matched volunteers who were admitted to our outpatient clinics. The exclusion criteria for the patients included in both groups were the following: acute left ventricular heart failure, acute coronary syndrome, chronic pulmonary hypertension, severe chronic obstructive lung disease, lung neoplasms, end-stage renal failure, sepsis, acute cerebrovascular disease, acute or chronic aortic dissection, and a history of neuropsychiatric disease. Even though the study was conducted during the COVID-19 pandemic, to obtain unbiased data, and due to a significant reduced number of COVID-19 patients with PE which were addressed to our clinic in the abovementioned period, we excluded those patients from both study groups. Thus, all study and control group patients presented a negative PCR-test for COVID-19 in the last 48 h before blood sample collection. To obtain a comprehensive medical history, detailed anamnesis was performed, and patients’ personal and hospital medical files were reviewed. After admission, a venous blood sample was collected from all patients, and was centrifuged at 3000 rpm for 15 min to separate plasma. Plasma BDNF levels were measured using Human BDNF ELISA based kits (Biovision, Milpitas, CA, USA), with a detection range between 80 pg/mL–16 ng/mL, an intra-assay reproducibility of CV < 10%, and an inter-assay reproducibility of CV < 12%. Echocardiography was performed using a General Electric Vivid^TM^ V7 ultrasound device (General Electric, Boston, CA, USA) to evaluate PE-specific ultrasound parameters, such as right ventricular diameter (RVd), tricuspid annular plane systolic elevation (TAPSE), and estimated systolic pulmonary arterial pressure (sPAP). Based on clinical and paraclinical parameters, pulmonary embolism severity index (PESI) and simplified PESI (sPESI) scores were determined for PE patients.

The study protocol was approved by the Ethics Committee of the Grigore T. Popa University of Medicine and Pharmacy, and by the Ethics Committee of the St. Spiridon Emergency Clinical Hospital. All research was conducted according to the ethical guidelines of the Declaration of Helsinki Principles, revised in 2013. All patients have signed a standard written informed consent to participate in this study.

### 2.2. Statistical Analysis

Statistical analysis of the data gathered in our study was performed using IBM SPSS Statistics for Windows v.26.0 (IBM, Armonk, NY, USA). The Kolmogorov–Smirnov test was used to assess the normal distribution of the continuous variables in the study population. Descriptive data for normally distributed continuous variables are reported as mean ± standard deviation (STD), together with the minimum and maximum value, and as medians with interquartile ranges (IQRs) for variables not normally distributed. Categorical variables are expressed as frequencies and percentages. The data from the study group and control group were compared using parametric (independent sample *t*-test) or non-parametric (Mann–Whitney U) tests. The correlation between variables in our study was measured using the Pearson correlation coefficient for continuous variables, or Spearman’s correlation coefficient for nonparametric variables. Binary logistic regression was used to estimate the influence of continuous predictors on binary outcomes. Quality assessment of the logistic regression model was performed using the Hosmer–Lemeshow test. Linear regression was conducted to observe how variables vary between each other. The diagnostic properties of BDNF and D-dimer for PE were estimated using receiver operating characteristic (ROC) analysis by comparing areas under the curve (AUC) for both BDNF and D-dimers. ROC analysis was also used to identify a cut-off value for BDNF in PE diagnosis. A *p*-value < 0.05 was considered statistically significant.

## 3. Results

### 3.1. General Charactersistics

Our study included a total of 145 patients, out of which, 90 patients with acute PE represented the study group, and 55 volunteers without PE represented the control group. Demographics such as age and sex distribution were similar between the two groups. Furthermore, after comparing the baseline characteristics between the patients with PE and the control group, we found no significant differences regarding the prevalence of the main risk factors for PE: high arterial blood pressure (*p* = 0.18), diabetes mellitus (*p* = 0.36), a history of recent surgery (*p* = 0.93), or active cancer (*p* = 0.08). As expected, clinical, paraclinical, and echocardiographic parameters specific for PE (heart rate, systolic blood pressure, peripheric oxygen saturation, leucocytes, C-reactive protein, RVd, and TAPSE) were significantly different in the study group (*p* < 0.05). The general characteristics of both groups are summarized in Table 1.

In the study group, 61 (67.8%) patients had an increased risk of 30-day mortality, as evaluated by sPESI score. According to the PESI risk stratification, the PE group presented 20 (22.2%) patients with a very low 30-day mortality risk (class I), nine patients (10%) with a low mortality risk (class II), 19 (21.1%) patients with a moderate mortality risk (class III), 19 (21.1%) patients with a high mortality risk (class IV), and 23 patients (25.6%) with a very high mortality risk (class V). The number of patients who required thrombolytic therapy was 21 (23.3%), whereas nine (10%) patients of the study group died within 30 days of admission. The risk profile of the PE group patients is synthesized in Table 2.

### 3.2. Biomarkers and BDNF Profile

As seen in Table 3, classical thrombosis and cardiac biomarkers such as D-dimers, high-sensitive troponin (hsTnI), and NT-proBNP were significantly increased in the study group (*p* < 0.01).

Interestingly, in the PE patient group, the median BNDF plasma levels were significantly decreased compared with the control group (403 vs. 644 pg/mL, *p* < 0.001).

Low levels of BDNF showed significant correlations with RVd values (*p* = 0.029) and NT-proBNP levels (*p* = 0.009), as a decrease in plasma BDNF was associated with an increase in RVd (r = −0.231) and NT-proBNP (r = −0.275). Furthermore, a similar decrease of BDNF was directly correlated with a decrease in TAPSE value (r = 0.254, *p* = 0.016). BDNF values did not correlate with clinical and paraclinical parameters such as age, sex, body mass index, blood pressure, heart rate, oxygen saturation, hemoglobin, leucocytes, thrombocytes, CRP, D-dimers, troponin, sPAP, RV/LV ratio, and LVEF. The correlations between BDNF and clinical and paraclinical parameters are shown in Table 4.

### 3.3. BDNF for Diagnosis of PE

**Figure 1:**
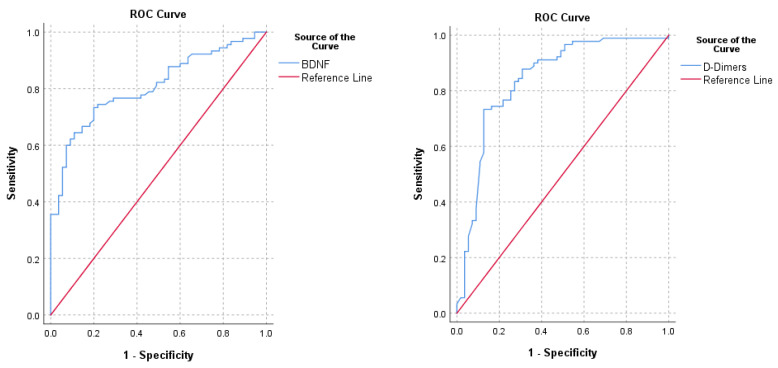
Receiving operating characteristics curves (ROC) for D-dimers and BDNF.

To evaluate the diagnostic potential of BDNF, we performed a ROC analysis, which presented an AUC of 0.807 (95% CI 0.738–0.876, *p* < 0.001), comparable with the AUC of D-dimers of 0.840 (95% CI 0.769–0.912, *p* < 0.001). The ROC curves for both BDNF and D-dimers are illustrated in Figure 1.

Analyzing the ROC curve for the diagnosis of PE, we found a cut-off value of BNDF of 564 pg/mL with a sensitivity of 74.4%, a specificity of 78.2%, a positive predictive value of 84.8%, and a negative predictive value of 65.15%. Furthermore, we performed a binary logistic regression to analyze the combination between BDNF levels and D-dimer levels for the diagnosis of pulmonary embolism. We found that one unit increase in D-dimers increased the probability of PE by 134%, whereas a one unit increase of BDNF decreased the probability of PE by 35% (data shown in Table 5).

### 3.4. BDNF for Risk Stratification of PE

**Figure 2:**
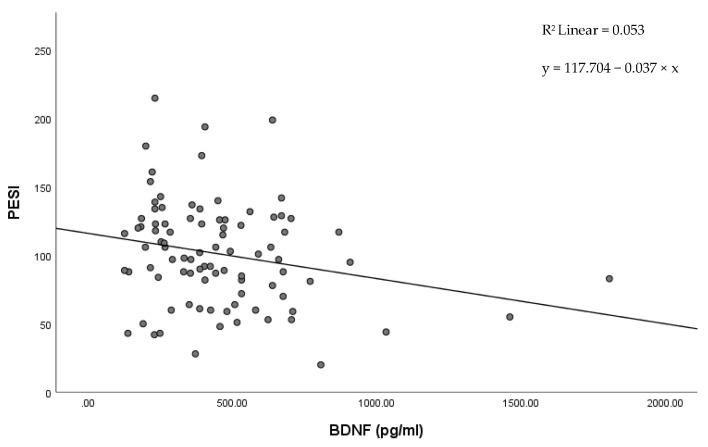
Scatter plot showing the correlation between BDNF and PESI score.

In our study, low BDNF levels correlated with previously validated predictors for the severity of PE. Low BNDF levels were correlated with an increased PESI score (r = 0.240, *p* = 0.023). The regression equation (Figure 2) was y = 117.704 − 0.037 × x with r^2^ = 0.053.

Furthermore, patients with a high risk of PE evaluated by sPESI and patients who required thrombolytic treatment presented overall significantly lower plasma levels of BDNF (*p* ≤ 0.01). Patients who died within 30 days of diagnosis showed significantly decreased BDNF expression (*p* = 0.05), as can be observed in Table 6. However, binary logistic regression analysis could not return a significantly statistical prediction model for mortality based on BDNF levels in our study group (*p* > 0.05).

## 4. Discussion

Acute PE is a condition with polymorphic clinical presentation, which can be challenging to diagnose. Furthermore, though this condition is associated with high mortality, risk stratification and the proper selection of patients for thrombolytic therapy may be difficult, especially in an emergency setting [3]. Recent evidence indicates that BDNF plays an important role in cardiovascular signaling, and may be used for risk stratification in various cardiac conditions [12]. In our study, we aimed to assess the expression of BDNF in the plasma of patients diagnosed with acute PE. The results were compared to control patients from the general population to evaluate the impact of BDNF on the diagnosis and the prognostic of PE.

In our research, the plasma levels of BDNF were significantly lower in the patients with acute PE. Additionally, ROC curve analysis showed a cut-off BDNF level of 564 pg/mL, with a specificity of 78.2%, a sensitivity of 74.4%, a positive predictive value of 84.8%, and a negative predictive value of 65.1% for predicting PE. The AUC value of the D-dimer ROC analysis was slightly higher when compared with BDNF. However, the confidence intervals of both AUC overlap, indicating that both D-dimers and BDNF had comparable diagnostic accuracies for PE. According to current guidelines, D-dimer assessment is the standard biomarker test recommended to improve the diagnosis of PE [3]. However, D-dimers have up to 96% sensitivity, but a low specificity, ranging from 41 to 70% [13]. Consequently, we hypothesized that BDNF can be used as an additional biomarker to improve the D-dimer diagnostic performance for PE. The binary logistic regression analysis validated that BDNF can be used together with D-dimers to create a diagnostic model for acute PE, showing that a BDNF level increase results in a 35% reduction of the probability of PE. Although the sample size of patients with PE and cancer was reduced in our study group, the BDNF levels were not significantly influenced in these patients. Additionally, several studies showed that BDNF levels were significantly increased, rather than decreased, in patients with various types of cancer, but without PE [14,15]. Taken together, the results from our study and the available data from the literature offer a new research hypothesis in which both D-dimer and BDNF testing may improve the pre-test probability of acute PE in a specific subpopulation, such as cancer patients.

The overall risk profile and mortality in our study population were similar with previous observational studies focusing on acute PE [16,17]. This allowed us to examine if BDNF levels may be used as predictors for PE evolution by comparing parameters already validated for risk stratification. Troponin and NT-proBNP are biomarkers reflecting acute right ventricular injury and dysfunction, as confirmed by several clinical studies in which increased plasma levels of these biomarkers are associated with a worse clinical outcome of PE [18,19,20]. In our study, decreased BDNF values were correlated with high NT-proBNP levels. There was no statistical correlation between BDNF and troponin. In addition to cardiac biomarkers, acute right ventricular failure in the context of PE can be assessed by echocardiographic parameters, such as RVd, sPAP, and TAPSE, or CTPA parameters, such as RV/LV ratio, or imagistic parameters, which, if modified, are associated with a worse outcome [21,22]. In our study group, we found statistically significant correlations between low BDNF levels, increased RVd, and decreased TAPSE, but there was no correlation with sPAP or RV/LV ratio. Growing evidence suggests that inflammation plays an important role in the physiopathology of PE. Various parameters reflecting systemic inflammation are usually increased in PE patients, and may be also associated with a poorer prognosis [23,24]. In our study, both leukocytes and CRP levels were increased in PE patients, but we did not find any correlation between these parameters and BDNF expression.

PESI score and its simplified version (sPESI) have been validated in multiple prospective studies, and are the most used risk predictors in clinical practice [25,26,27]. Our research revealed a decreased BDNF plasma expression in the high-risk sPESI patients compared to the low-risk sPESI patients. These results are supported by the linear regression between the decreased BDNF levels and the increased PESI score.

We further evaluated if there were any significant differences of BDNF expression in hemodynamic unstable patients who required thrombolysis, and in patients who died within 30 days of admission. Thrombolysis is a high-risk therapy associated with an increased incidence of adverse effects, such as severe hemorrhage, and it is recommended in PE patients who have a very high mortality risk [28]. Interestingly, the BDNF values in the patients requiring thrombolysis were significantly lower when compared with patients without thrombolysis, and, similarly, the BDNF values were lower in patients who died within 30 days. However, the reduced number of patients and adverse events did not allow us to use ROC analysis for the prediction of mortality. Moreover, binary logistic regression could not validate a probability model for the same endpoint. The correlation of low BDNF levels with prognostic parameters, thrombolysis, and mortality found in our study raises an interesting hypothesis in which plasma BDNF may be used for risk assessment in PE patients.

To the best of our knowledge, the relationship between BDNF activity and PE has not been evaluated in experimental or clinical studies. To characterize the relationship between BDNF expression and the physiopathology of PE, we focused on two major pathological events during PE, i.e., right ventricular dysfunction and pulmonary hypoxia.

Several studies have shown that in the normal heart, BDNF may be a modulator of contractility and relaxation via Ca^2+^/calmodulin-dependent protein kinase II signaling [29,30]. Additionally, BDNF increases the exercise capacity in heart-failure mice due to the enhancement of fatty acid oxidation via the activation of the AMPKα-PGC1α pathway [31]. However, during ventricular dysfunction, both plasma and serum BDNF levels are reduced, and a low BDNF concentration was positively correlated with heart failure severity and prognosis [32,33,34]. These results were further confirmed by Bahls et al., who reported that low levels of BDNF are correlated with increased NT-proBNP in heart-failure patients [35]. The mechanism involved in the decreased circulating BDNF levels in heart failure is not well known, but it has been assumed that decreased heart cell mass is associated with reduced BDNF production. Likewise, the overactivation of the sympathetic nervous system during heart failure may increase the circulating glucocorticoid concentration, which, in turn, reduces BDNF release from the hippocampus [33]. Compared to the aforementioned studies, our research evaluated acute right ventricular dysfunction during PE instead of left ventricular dysfunction, but we might assume that the molecular mechanism of BDNF expression may be similar. Our hypothesis is based on the correlation between parameters reflecting right ventricular dysfunction, such as high RVd, low TAPSE, high NT-proBNP, and low levels of BDNF. This may suggest that right ventricular heart failure might be responsible for the low level of BDNF expression. However, to confirm this theory, further experimental and observational studies are required.

Another pathophysiological mechanism involved in PE is represented by the mismatch in pulmonary ventilation and perfusion, which leads to hypoxia. Kwapiszweska et al. showed that BDNF levels are increased in hypoxic mouse lung specimens, as well as in samples from the lung tissues of patients with idiopathic pulmonary arterial hypertension. During lung hypoxia, BDNF induces pulmonary artery smooth muscle cell proliferation through the BDNF-TrkB-ERK1/2 pathway [36]. Likewise, Helan et al. showed that in the context of hypoxia, human pulmonary artery endothelial cells secrete BDNF via the hypoxia inducible factor 1 alpha pathway, leading to NO production [11]. Although BDNF levels are increased in the tissue samples of hypoxic lungs, further in vivo studies are required to establish if BDNF plasma concentrations are also modified in acute or chronic pulmonary hypoxia.

Low plasma levels of BDNF have previously been reported in patients suffering from neuropsychiatric diseases such as bipolar disease, schizophrenia, and depression [37]. In general, cardiovascular diseases, including pulmonary embolism, are associated with different forms of depressive disorders [38,39]. Several studies identified that decreased levels of BDNF in the context of inflammation can affect neuroplasticity and increase the susceptibility of developing depression in patients with coronary artery disease [40,41]. Nonetheless, in our study, we did not include patients with a history or showing symptoms of neuropsychiatric disease; therefore, we considered that low BNDF levels were independent of these conditions.

### Limitations of the Study

Our study has several limitations. The population sample included in our study was relatively small and the exclusion criteria make it difficult to extrapolate to all patients with PE, especially in those with concomitant severe morbid conditions. However, the risk profile distribution and mortality rates were similar to those from other published studies. Although we measured BDNF immediately after admission, evaluating the changes of BDNF expression over the long term may offer further insights into its pathophysiologic role in PE. Nevertheless, to the best of our knowledge, this study is the first one which evaluated plasma BDNF expression in patients with PE.

## 5. Conclusions

Our study revealed that the expression of plasma BDNF is significantly decreased in patients with PE compared with controls from the general population. BDNF levels lower than 564 pg/mL presented a sensitivity of 74.4%, a specificity of 78.2%, a positive predictive value of 84.8%, and a negative predictive value of 65.15% for PE diagnosis in this study cohort. Interestingly, BDNF expression also correlated with risk predictors for severe outcomes in PE, such as NT-proBNP, RVd, TAPSE, PESI score, the need of thrombolytic treatment, and mortality.

These results suggest that BNDF may be considered as a promising additional biomarker used in the management of patients with PE. Together with D-dimers, BDNF could significantly improve the pre-test probability and reduce the rate of false-positive results. Furthermore, low BNDF levels have also been associated with a poor outcome, and may be considered as an additional risk marker in PE.

Clearly, our results should be validated in further experimental studies focusing on the precise mechanisms of BDNF expression in PE. Moreover, larger patient cohort studies comparing BDNF level expression in PE patients, as well as in patients with other acute chest syndromes, such as acute coronary syndrome, COPD, pulmonary infections, and aortic dissections, are mandatory. These studies may help to establish the exact pathophysiological and clinical role of BDNF in PE.

## Figures and Tables

**Table 1:** General characteristics of the study groups.

Characteristics	Total (*n* = 145)			Pulmonary Embolism (*n* = 90)			Study Group (*n* = 55)			*p*-Value
	Min	Mean ± STD	Max	Min	Mean ± STD	Max	Min	Mean ± STD	Max	
Age (years)	18	61.6 ± 14.30	91	18	62.83 ± 14.49	90	29	59.5 ± 13.84	91	*p* = 0.182
Gender (N, %)	Male 82 (56.55%)	Female 63 (43.44%)		Male 51 (56.67%)	Female 39 (43.33%)		Male 31 (56.36%)	Female 24 (43.63%)		*p* = 0.554
**Systolic blood pressure (mmHg)**	50	127.40 ± 21.53	200	50	122.03 ± 22.26	200	110	136.69 ± 16.67	185	***p* < 0.001**
**Diastolic blood pressure (mmHg)**	18	66.06 ± 13.83	110	18	62.83 ± 14.49	91	60	71.36 ± 10.86	110	***p* < 0.001**
**Heart rate (bpm)**	50	88.48 ± 19.71	150	53	95.41 ± 19.88	150	50	77.11 ± 13.60	111	***p* < 0.001**
**Oxygen saturation in ambient air (%)**	81	94.84 ± 4.89	100	81	92.05 ± 4.19	99	97	99.41 ± 0.78	100	***p* < 0.001**
Surgery within 30 days		11 (7.59%)			7 (7.78%)			4 (7.27%)		*p* = 0.936
Active cancer		13 (8.96%)			11 (12.2%)			2 (3.63%)		*p* = 0.089
Post-partum		2 (1.38%)			1 (1.11%)			1 (1.81%)		*p* = 0.715
Diabetes mellitus		29 (20%)			16 (17.77%)			13 (23.63%)		*p* = 0.365
Arterial hypertension		69 (47.58%)			47 (52.22%)			22 (40%)		*p* = 0.184
Smoking		46 (31.72%)			33 (36.67%)			13 (23.63%)		*p* = 0.152
BMI (kg/m^2^)	17.16	26.06 ± 3.79	40.4	17.16	26.32 ± 4.32	40.4	20.31	25.64 ± 2.68	36.45	*p* = 0.303
**Leucocytes (×10^9^/L)**	4.06	9.75 ± 3.57	23.9	4.06	10.32 ± 4.22	23.9	5.11	8.81 ± 1.78	15.33	***p* = 0.013**
Hemoglobin (g/L)	9.2	13.29 ± 1.58	17.9	9.2	13.17 ± 1.77	17.9	9.2	13.48 ± 1.19	17	*p* = 0.249
Thrombocytes (×10^9^/L)	93	253.51 ± 100.20	745	96	253.45 ± 120.16	745	93	253.61 ± 54.55	415	*p* = 0.992
**CRP (mg/dL)**	0.02	5.37 ± 11.77	118	0.08	8 ± 14.29	118	0.02	1.08 ± 1.74	11	***p* < 0.001**
Glucose (mg/dL)	70	113.43 ± 33.53	310	80	117.19 ± 39.34	310	70	107.36 ± 19.84	160	*p* = 0.09
Creatinine (mg/dL)	0.42	0.97 ± 0.38	3.71	0.42	0.97 ± 0.43	3.71	0.6	0.98 ± 0.27	1.77	*p* = 0.796
LVEF (%)	15	51.28 ± 7.43	65	15	51.4 ± 7.3	65	35	51.09 ± 0.98	65	*p* = 0.809
**RV diameter (mm)**	22	32.69 ± 6.69	50	24	35.7 ± 6.48	50	22	27.7 ± 3.25	35	***p* < 0.001**
**TAPSE (mm)**	11	20.07 ± 4.15	30	11	18.23 ± 4.03	30	19	23.09 ± 2.03	28	***p* < 0.001**
sPAP (mmHg)	10	26.08 ± 9.63	58	10	28.27 ± 11.05	58	15	22.49 ± 5.03	35	*p* = 0.232
**RV/LV ratio**		N/A		≤1	>1			N/A		***p* < 0.01**
				56 (62.2%)	34 (37.8%)					

BMI—body mass index, SBP—systolic blood pressure, HR—heart rate, CRP—C-reactive protein, RV—right ventricle, LV—left ventricle, TAPSE—tricuspid annular plane systolic elevation, sPAP—systolic pulmonary artery pressure, LVEF—left ventricle ejection fraction, PESI—pulmonary embolism severity index, STD—standard deviation, N/A—not applicable, Bold font indicates statistical significance.

**Table 2:** Risk profile of the PE study group.

Characteristics	PE Patients				
PESI score	Min.	Mean ± STD			Max.
	20	101.13 ± 38.14			215
PESI stratification	Class I	Class II	Class III	Class IV	Class V
	20 (22.2%)	9 (10%)	19 (21.1%)	19 (21.1%)	23 (25.6%)
sPESI	<1		≥1		
	29 (32.2%)		61 (67.8%)		
Thrombolysis	No		Yes		
	69 (76.7%)		21 (23.3%)		
Death in 30 days	No		Yes		
	81 (90%)		9 (10%)		

PESI—pulmonary embolism severity index, sPESI —simplified pulmonary embolism severity index, STD—standard deviation.

**Table 3:** Biomarker profiles in the study population.

Biomarker	PE Group (*n* = 90)	Control Group (*n* = 55)	*p*-Value
D-dimers (µg/mL)	5.1 (3.05–5.23)	1.27 (0.67–3.12)	*p* < 0.001
hsTnI (ng/L)	24.5 (5.45–75.75)	4.83 (1.23–12)	*p* < 0.001
NT-proBNP (pg/mL)	1482.5 (239–3255)	86.3 (30–150)	*p* < 0.001
BDNF (pg/mL)	403 (252–582)	644 (576–784)	*p* < 0.001

hsTnI—high-sensitive troponin; NT-proBNP—N-terminal pro b-type natriuretic peptide; BDNF—brain-derived neurotrophic factor. Values are expressed as medians (IQR—interquartile range).

**Table 4:** Correlations between BDNF levels and clinical and paraclinical parameters in the PE group.

Parameter	BDNF	
	r	*p*-Value
Age	0.044	0.681
Sex	−0.119	0.263
BMI	−0.052	0.627
Cancer	−1.04	0.211
SBP	0.069	0.521
HR	−0.162	0.130
Oxygen saturation	0.076	0.479
Hemoglobin	−0.100	0.350
Leukocytes	−0.166	0.117
Thrombocytes	−0.172	0.104
CRP	−0.155	0.143
**RVd**	**−0.231**	**0.029**
**TAPSE**	**0.254**	**0.016**
sPAP	−0.127	0.232
LVEF	0.126	0.235
hs cTnI	−0.038	0.723
D-dimers	−0.48	0.651
**NT-proBNP**	**−0.275**	**0.009**
RV/LV ratio > 1	−0.154	0.149

BMI—body mass index, SBP—systolic blood pressure, HR—heart rate, CRP—C-reactive protein, RVd—right ventricle diameter, RV—right ventricle, LV—left ventricle, TAPSE—tricuspid annular plane systolic elevation, sPAP—systolic pulmonary artery pressure, LVEF—left ventricle ejection fraction, PESI—pulmonary embolism severity index, NT-proBNP—amino-terminal pro-brain natriuretic peptide, hs cTnI—high sensitivity troponin I, r—correlation coefficient, Bold font indicates statistical significance.

**Table 5:** Binary logistic regression of BDNF and D-dimers for diagnostic of PE.

Biomarker	Odds Ratio	95% CI for Exp(B)		*p*-Value
		Lower	Upper	
D-dimers	2.34	1.75	3.14	*p* < 0.01
BDNF	0.65	0.53	0.79	*p* < 0.01

**Table 6:** BDNF profile according to risk category.

Biomarker	sPESI < 1 (*n* = 29)	sPESI ≥ 1 (*n* = 61)	*p*-Value	Standard Therapy (*n* = 69)	Thrombolytic Therapy (*n* = 21)	*p*-Value	Survivors (*n* = 81)	Non-Survivors (*n* = 9)	*p*-Value
BDNF	515 (360–690)	386 (233–483)	0.009	441 (311–635)	263 (223–454)	0.01	423 (259–628)	283 (232–430)	0.05

## Data Availability

The data presented in this study are available within the article.
